# Industrial arsenic contamination causes catastrophic changes in freshwater ecosystems

**DOI:** 10.1038/srep17419

**Published:** 2015-11-30

**Authors:** Guangjie Chen, Haibin Shi, Jianshuang Tao, Li Chen, Yuanyuan Liu, Guoliang Lei, Xiaohai Liu, John P. Smol

**Affiliations:** 1 Key Laboratory of Plateau Lake Ecology and Global Change, School of Tourism and Geography, Yunnan Normal University, Kunming, Yunnan, China; 2 State Key Laboratory of Subtropical Mountain Ecology (Funded by Ministry of Science and Technology and Fujian Province), College of Geographical Sciences, Fujian Normal University, Fuzhou, Fujian, China; 3 Yunnan Institute of Environmental Sciences, Kunming, Yunnan, China; 4 Paleoecological Environmental Assessment and Research Lab, Department of Biology, Queen’s University, Kingston, Ontario, Canada

## Abstract

Heavy metal pollution is now widely recognized to pose severe health and environmental threats, yet much of what is known concerning its adverse impacts on ecosystem health is derived from short-term ecotoxicological studies. Due to the frequent absence of long-term monitoring data, little is known of the long-tem ecological consequences of pollutants such as arsenic. Here, our dated sediment records from two contaminated lakes in China faithfully document a 13.9 and 21.4-fold increase of total arsenic relative to pre-1950 background levels. Concurrently, coherent responses in keystone biota signal pronounced ecosystem changes, with a >10-fold loss in crustacean zooplankton (important herbivores in the food webs of these lake systems) and a >5-fold increase in a highly metal-tolerant alga. Such fundamental ecological changes will cascade through the ecosystem, causing potentially catastrophic consequences for ecosystem services in contaminated regions.

Increasing exposure to heavy metal pollution is one of the biggest concerns for public health, water quality and ecosystem conservation^1^,^2^. Arsenic pollution in particular has recently attracted attention due to contamination of drinking water^3^,^4^. In contrast, human-caused arsenic contamination of surface waters and its ecological risks have received far less attention, despite the fact that ongoing industrialization and urbanization can significantly increase arsenic exposure of humans and ecosystems at large spatial and temporal scales^5^,^6^.

Ecotoxicological and bioaccumulation effects of arsenic pollution have been largely explored through experimental tests and short-term surveys^7^,^8^, but little is known of its long-term impacts on freshwater ecosystem health. Moreover, short-term experimental and monitoring studies cannot easily account for the cumulative and legacy effects of heavy metal exposure^9^,^10^. For such insights, long-term community-level responses must be assessed, which take into account pre-impact reference conditions. Unfortunately, due to the lack of long-term monitoring data, such data are rarely available, especially in areas such as China where industrial activity has increased rapidly with insufficient environmental oversight^11^,^12^.

Algae and invertebrates typically serve as the base of lacustrine food webs and therefore any assemblage change in response to heavy metal contamination can significantly alter the structure and functioning of freshwater ecosystems^13^,^14^. Here we focused on the production and community structure of algae and zooplankton, which are identifiable in sediments and can provide information on long-term environmental trends necessary to put any recent ecological changes into an appropriate temporal perspective^15^. Diatoms are an important group of freshwater algae characterized by high species diversity, short life cycle and strong sensitivities to heavy metal pollution^16^. Crustacean zooplankton are important herbivores playing a key role in lake food webs. Importantly, Cladocera are often prime ecotoxicological indicators for metal assessments, as many daphniids and bosminids are sensitive to heavy metal exposure^17^,^18^,^19^. Because both diatoms (species-specific siliceous valves) and Cladocera (chitinous body parts) are well preserved in lake sediments, they are also powerful and reliable paleolimnological indicators^15^.

Here, we present highly-resolved and well-dated paleolimnological records of two lakes from Southwest China with documented histories of arsenic contaminations. Yangzong, a large and deep lake (Fig. 1a and Supplementary Table 1), has experienced an industrial tailing leakage accident along the southwest part of the lake basin with arsenic concentrations in water increasing from ~7.3 μg L^−1^ in 2007 to ~177 μg L^−1^ in December 2008^20^. The arsenic level remained at ~50.0 μg L^−1^ in 2013 (Fig. 1b), well above the WHO standard level of 10.0 μg L^−1^ for drinking water. Meanwhile, shallow Datun Lake (Supplementary Table 1) has been susceptible to wastewater input during flooding periods as the south basin, which was dammed in 1966, has served as the dumping site for mineral tailings. The monitored arsenic concentrations in water spiked from ~12.0 μg L^−1^ in 1990 to ~1,140 μg L^−1^ in 2008. These numbers are especially alarming as arsenic concentrations as low as <100 μg L^−1^ can cause adverse effects on aquatic biota such as algae and invertebrates^21^, through damaging algal cell growth, subdivision and photosynthesis^16^ and inhibiting the development and reproduction of invertebrates^8^. Here we explore whether known arsenic pollution has had discernable and directional effects on key aquatic biota, using a long temporal perspective made available by sedimentary archives.

## Results

### History of industrial arsenic contaminations

**Figure 1:**
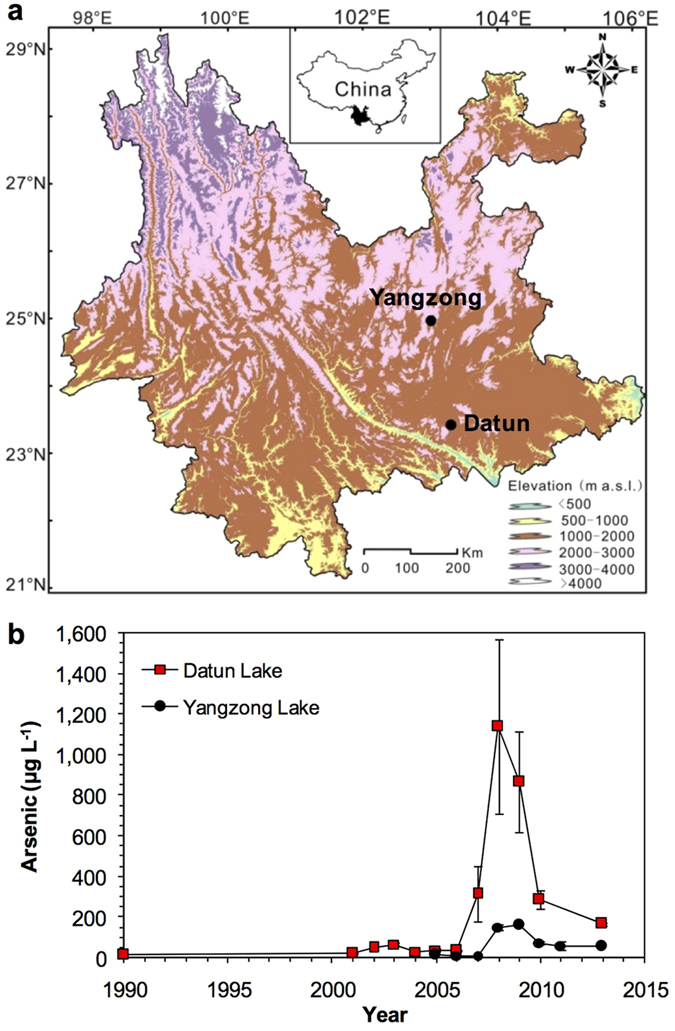
L Locations (**a**) and monitored lake water arsenic concentrations (**b**) of the two study sites from Yunnan, China. ake water arsenic data (annual mean ±1 standard error) were analyzed by the Yunnan Institute of Environmental Sciences, Kunming, China. The x-axis of the bottom panel is in calendar years and the site map was created using ArcMap10.0 (ESRI).

**Figure 2:**
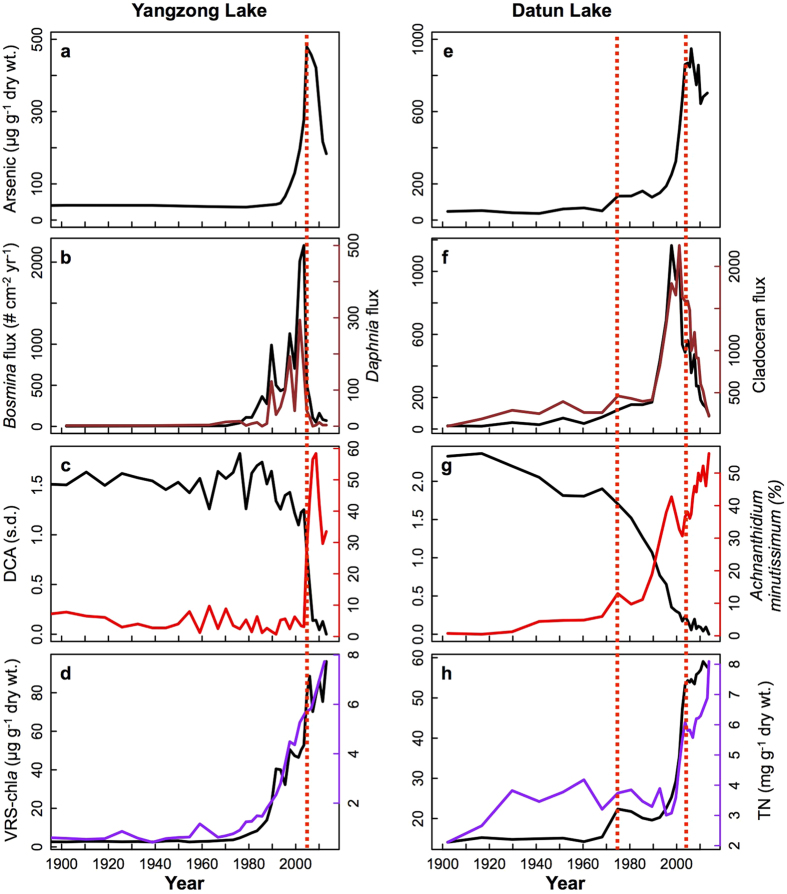
Multiple-proxy sediment records of the two study lakes from Yunnan, China, tracking limnological changes over the last century. (**a**,**e**) Total arsenic concentrations of sediment samples; (**b**,**f**) Fluxes of *Bosmina* (black) and *Daphnia* (brown, in Datun Lake the total cladoceran flux is shown as *Daphnia* was present in only six out of the 30 samples); (**c**,**g**) Gradient length of sediment diatom composition based on detrended correspondence analysis (DCA axis one score, black) and relative abundance of benthic diatom *Achnanthidium minutissimum* (red); (**d**,**h**) Stratigraphies of visible reflectance spectroscopy (VRS) chlorophyll *a* concentrations (black) and total nitrogen (TN) content (purple). The x-axis denotes sediment age in calendar years as estimated by ^210^Pb analysis (Supplementary Fig. 4) and the red dashed lines show the timings of significant industrial arsenic discharges at Yangzong Lake and Datun Lake.

Geochemical analyses clearly identified significant enrichment of arsenic in the sediments of both contaminated lakes, a trend that parallels the water monitoring records (Figs 1b and 2). During the pre-1950 period, sediment arsenic levels have remained stable with a mean value of 34.4 ± 5.1 (±1 SD) μg g^−1^ dry weight at Yangzong Lake and of 44.2 ± 7.1 (±1 SD) μg g^−1^ dry weight at Datun Lake (Fig. 2a,e). Thereafter, at around 2007, total arsenic concentrations spiked with a peak value of 479 and 949 μg g^−1^ dry weight, respectively (i.e.13.9 and 21.4 times pre-1950 background levels). It is likely that even these striking increases are conservative estimates of arsenic pollution because sediment records likely underestimate the lakewater arsenic levels (Fig. 1b), as only about 40–90% of metal input are typically retained in lake sediments^22^,^23^. A lack of a regional pattern of synchronous changes in arsenic among our study lakes and neighboring systems^24^ confirms that atmospheric inputs^25^,^26^ could not explain the striking arsenic spikes in our affected lakes.

Meanwhile, records of other heavy metals showed limited enrichment of sediment concentrations over the past century (Supplementary Fig. 1), suggesting their minor role in ecosystem change compared to the striking arsenic increases. When setting the pre-1950 levels of heavy metals as the background values in our lakes (Supplementary Fig. 1), the enrichment factors for both lead and zinc are less than 2–4 at Yangzong and Datun lakes and are significantly lower when compared to those of arsenic. The regional pattern of atmospheric input of lead and zinc was evidenced in neighboring lakes from our study area, particularly since the 1980 s^27^,^28^. In contrast to the changes in sediment arsenic concentrations, sediment profiles of other heavy metals (i.e. Cr, Co, Ni and Cu) generally showed a decreasing trend in both arsenic-contaminated lakes for the past century (Supplementary Fig. 1). We therefore conclude that the ecological risk associated with arsenic pollution in our contaminated lakes is overwhelming when compared to those of other major heavy metals.

### Biological responses

Zooplankton populations were clearly affected by the arsenic pollution. Over the 20^th^ century, there was a steady increase in the production of *Daphnia* and *Bosmina*, two key cladoceran herbivores, which closely followed nutrient enrichment (i.e. total nitrogen; Fig. 2d,h). In Yangzong Lake, cladoceran accumulation rates increased from 1.8 and 2.5 individuals cm^−2^ yr^−1^ in the early 1900’s to ~294 and 2,013 individuals cm^−2^ yr^−1^ by ~2000, respectively. However, despite the continuing trend of nutrient enrichment (Fig. 2d,h), zooplankton were decimated following the arsenic increase, with a >10-fold decline in population size (Fig. 2b,f). Specifically, the daphniid and bosminid fluxes dropped abruptly to 3.1 and 70.1 individuals cm^−2^ yr^−1^ ~2013 in Yangzong Lake, respectively, with a striking loss of bosminids from a peak value of 1,164 individuals cm^−2^ yr^−1^ in ~1998 to 87.3 individuals cm^−2^ yr^−1^ in ~2013 in Datun Lake.

Similar to the invertebrate record, our sedimentary diatom profiles tracked significant assemblage changes concurrent with the increase in arsenic concentrations. Diatom assemblage composition displayed an abrupt shift around the mid-2000 s in Yangzong Lake and a more gradual but unidirectional change since the 1970 s in Datun Lake (Fig. 2c,g), both of which closely track the arsenic trajectories (Pearson product moment correlation between arsenic and detrended correspondence analysis (DCA) axis 1 score of diatom composition = 0.69 and 0.86 respectively, *P* < 0.001). Specifically, the relative abundance of benthic species such as *Achnanthidium minutissimum* increased consistently from <10% to >50% in both contaminated lakes and was the main driver of the diatom assemblage changes (i.e. DCA axis 1 score; *r* = 0.91 and 0.96 respectively, *P* < 0.001). *A. minutissimum* is known to be highly resistant to metal contamination in freshwaters^16^,^29^ and can proliferate in high arsenic concentrations^21^. Meanwhile, taxa such as *Fragilaria construens* and *F. crotonensis* cannot survive at high arsenic levels^29^ and their populations were either extirpated or strongly declined (Supplementary Fig. 2).

## Discussion

Although the detrimental effects of heavy metal pollutants such as arsenic have been identified in ecotoxicological experiments^8^,^21^,^30^, there has thus far been little evidence on this metal’s long-term ecological consequences at the community level. Our empirical records showed highly synchronous changes in both keystone zooplankton and diatom communities, signaling that critical ecological transitions had occurred in response to industrial arsenic contamination. The legacy effect of arsenic contamination was also evident in the lack of community recovery in both algae and zooplankton, despite declining arsenic levels more recently in our lakes (Figs 1 and 2). Therefore, this retrospective analysis, using lake sediments, provides a unique perspective on long-term ecosystem health that is beyond the scope of short-term studies. Significant loss of herbivores and strongly altered algal composition, as a result of arsenic toxicity, can severely alter ecosystem health and stability^31^. In addition, arsenic assimilated by aquatic organisms can also be transferred through food digestion along food webs^7^,^32^. For example, it was found that the threshold level of arsenic sublethal toxicity through dietary metals can be lower for consumers than that of dissolved metals^31^,^32^. Therefore, arsenic contamination can also impact the structure of lake food webs through trophic transfer.

Eutrophication was also an obvious stressor in our study lakes during the last few decades; however, it cannot account for the abrupt ecosystem shift in our arsenic-polluted lakes. Contrasting to the pattern of community shift triggered by planktonic diatoms due to lake eutrophication in this study region^33^, the major change of diatom assemblages in Yangzong and Datun was predominantly associated with metal-tolerant benthic taxa (Fig. 2c,g and Supplementary Fig. 2). Furthermore, this nutrient enrichment resulted in increased zooplankton populations (Fig. 2b,f) up until arsenic concentrations reached a mean of over ~100 μg L^−1^ in the water of our impacted lakes (Fig. 1b), which resulted in the decimation of secondary producers. Therefore, nutrient enrichment had played an important role in affecting lake communities, but its role was overridden by the detrimental effects of arsenic contamination when a threshold level of arsenic concentrations was reached. Interestingly, past algal production (estimated via sedimentary Chl*a*; Fig. 2d,h), as well as other proxies for lake production (i.e. organic matter and total carbon contents; Supplementary Fig. 1), all displayed an increasing trend in the sediment cores despite the high levels of arsenic. This suggests that some autotrophs such as *Achnanthidium minutissimum*, as evidenced in our lakes, and submerged macrophytes^20^,^34^ can still thrive, and that the loss of cladoceran herbivores likely freed certain metal-tolerant algae from the negative effects of grazing^14^.

**Figure 3:**
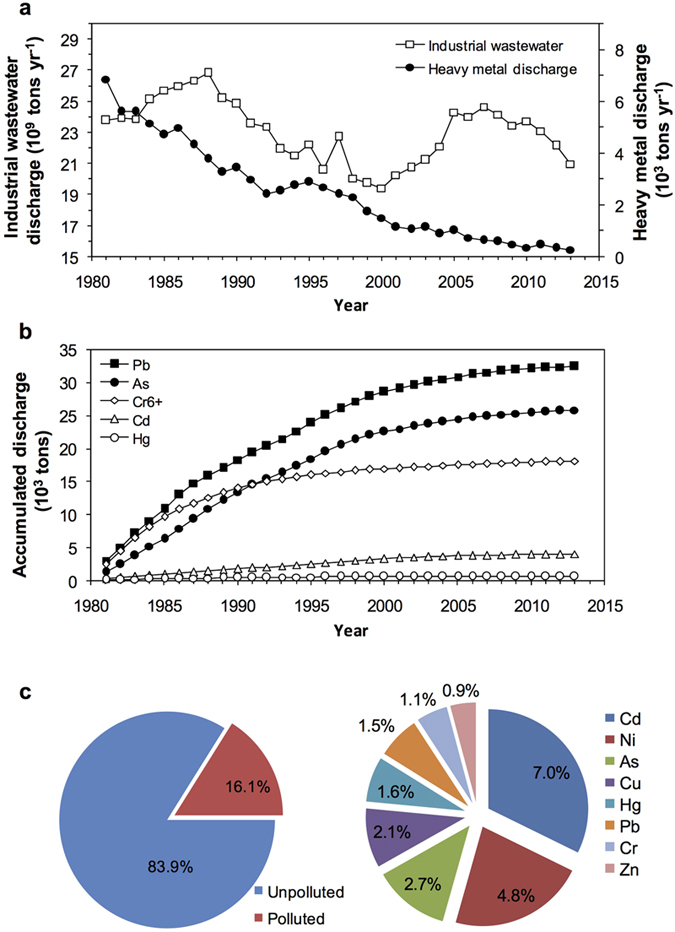
Time series of annual industrial wastewater and heavy metal discharges during 1981–2013 (**a**), accumulated discharge of heavy metals derived from industrial wastewaters (**b**) and soil pollution status (**c**) in China^44^. The industrial wastewater and heavy metal discharge data for 1991–2013 were collated from the annual environmental reports of the Ministry of Environmental Protection (URL http://www.zhb.gov.cn/zwgk/hjtj/nb/, accessed January, 2015). The national soil survey data (**c**) show the proportion of total soil sample sites polluted according to the Chinese national soil quality standards (GB15618–1995), covering a land area of 6.3 million km^2^, and those of the surveyed sites polluted with each of the eight heavy metals across China. The National Soil Pollution Survey Bulletin was jointly released by the Ministry of Environmental Protection and the Ministry of Land Resources in April 2014 (URL http://www.zhb.gov.cn/gkml/hbb/qt/201404/t20140417_270670.htm, date of access:17/04/2014). The x-axes of data panels (**a**,**b**) are in calendar years.

The threat of arsenic contamination derived from wastewater discharge is widespread in many industrial and urban areas around the world^1^,^11^. For example, in China alone, industrial wastewater discharge fluctuated annually in the range of 20–27 billion tonnes during 1981–2013 (Fig. 3a). Despite a declining trend in annual heavy metal loadings, the accumulated amount has reached >25 thousand tonnes of arsenic during the last three decades (Fig. 3b). These large stocks of pollutants, if not properly treated, can severely damage environmental quality (Fig. 3c) and ecosystem health. However, comprehensive monitoring has rarely accompanied these pollution events in the past, and so only indirect forensic approaches, like those used in this study, can supply these critical data. Remarkably altered algal composition and major losses of keystone herbivores indicate a severely threatened food web structure due to arsenic contamination, and thus will have far-reaching consequences for ecosystem services as these changes cascade through the ecosystem.

## Methods

### Sediment collection and dating

A Renberg gravity corer^35^ was used for collecting sediment cores at the central basin in each of the two lakes in 2013. Each core was sub-sampled at 0.5-cm and 1-cm intervals, respectively, for the top 5 cm and the rest of the core. Sub-samples were kept in the dark and refrigerated before being freeze-dried in the laboratory. Chronological sequences were constructed based on the radioactivities of bulk sediment ^210^Pb and ^137^Cs. The radiometric measurements of total ^210^Pb and ^226^Ra were collected using a Canberra well-detector gamma spectrometer (GCW3023) at Yunnan Normal University. The unsupported ^210^Pb values were used for constructing the model of Constant Supply of Rate (CRS) after subtracting the activities of ^226^Ra from those of total ^210^Pb^36^.

### Sediment metal concentrations

Freeze-dried samples were grounded in an agate mortar and then digested in a microwave digestion system with a HNO_3_-HF-HClO_4_-HCl acid mixture solution. Heavy metal concentrations were measured on an inductively coupled plasma-mass spectrometry (ICP-MS) instrument (X series II, Thermo, USA) at Fujian Normal University. QA/QC was performed through running certified reference samples, internal standards, blanks and duplicates with every batch of 20 samples. National standard lake sediment samples of China (GBW 07309) were measured with an analytical precision of ±5% of certified values. Parallel analyses of mixed solutions containing Rh, In and Re at 5 μg L^−1^ concentration were performed as internal standards with a recovery rate of 85–115%. Replicate analyses of sediment samples resulted in analytical uncertainties of <5%.

### Sediment nutrients and lake production

Homogenized bulk samples were measured for total nitrogen (TN), total carbon (TC) and the C/N mass ratio through an automated FLASH HT Plus elemental analyzer with a precision of ±1.0%, following standard methods^37^. Lake primary production was estimated from sediment chlorophyll *a* concentrations using visible reflectance spectroscopy^38^. This approach tracks the primary chlorophyll *a* pigment, plus all chlorophyll *a* isomers and degradation products, including pheophytin *a* and pheophorbide *a*, which are collectively referred to as Chl*a*. Sediment pigment concentrations were extracted in the unit of μg g^−1^ dry weight. Organic matter contents were measured by loss on ignition (LOI) at the temperatures of 550 °C for 2 h using a muffle furnace.

### Biological analyses

Sediment samples were processed for diatom analysis following standard methods^39^. A minimum of 400 diatom valves was identified for each sample and counted along transects at a 1,000× magnification with a Leica DM 2500 microscope with differential interference contrast and phase contrast optics. We primarily followed the diatom nomenclature and taxonomy of Krammer and Lange-Bertalot^40^.

We followed standard methods^41^ to prepare sedimentary cladoceran samples. For identifying and enumerating subfossil cladocerans, the slides were scanned and counted using a compound microscope under 200× and 400× magnifications. At least 100 cladoceran individuals were enumerated or at least 10 slides were full scanned for each sample. We mainly used Alonso^42^ as the taxonomic guides for our identifications. To take into account differential sedimentation rates, the count was then recalculated as a flux rate (individuals cm^−2^ year^−1^) to approximate the secondary production in this study (Supplementary Fig. 3). *Daphnia* were identified to species group (*Daphnia longispina* spp.), as post-abdominal claws were the only remains found in sediment.

### Statistical methods

Detrended Correspondence Analysis (DCA) was applied to extract the maximum direction in the diatom assemblage change in SD unit^33^. All data were tested for normality before further analyses (i.e. correlation analysis) were conducted. The software of R with basic and vegan packages^43^ was applied for all the statistical analyses.

## Additional Information

**How to cite this article**: Chen, G. *et al.* Industrial arsenic contamination causes catastrophic changes in freshwater ecosystems. *Sci. Rep.* **5**, 17419; doi: 10.1038/srep17419 (2015).

## Supplementary Material

Supplementary Information
