# Supplementary material for: Industrial arsenic contamination causes catastrophic changes in freshwater ecosystems
Source: Sci Rep. 2015 Nov 30;5:17419. doi: 10.1038/srep17419 (PMC4663503; doi:10.1038/srep17419)
Supplement: Supplementary Information [file srep17419-s1.pdf]

Supplementary Information

Industrial arsenic contamination causes catastrophic changes in freshwater ecosystems

Guangjie Chen[1]*, Haibin Shi[1], Jianshuang Tao[1], Li Chen[1], Yuanyuan Liu[1], Guoliang Lei[2], Xiaohai Liu[3], John P. Smol[4]

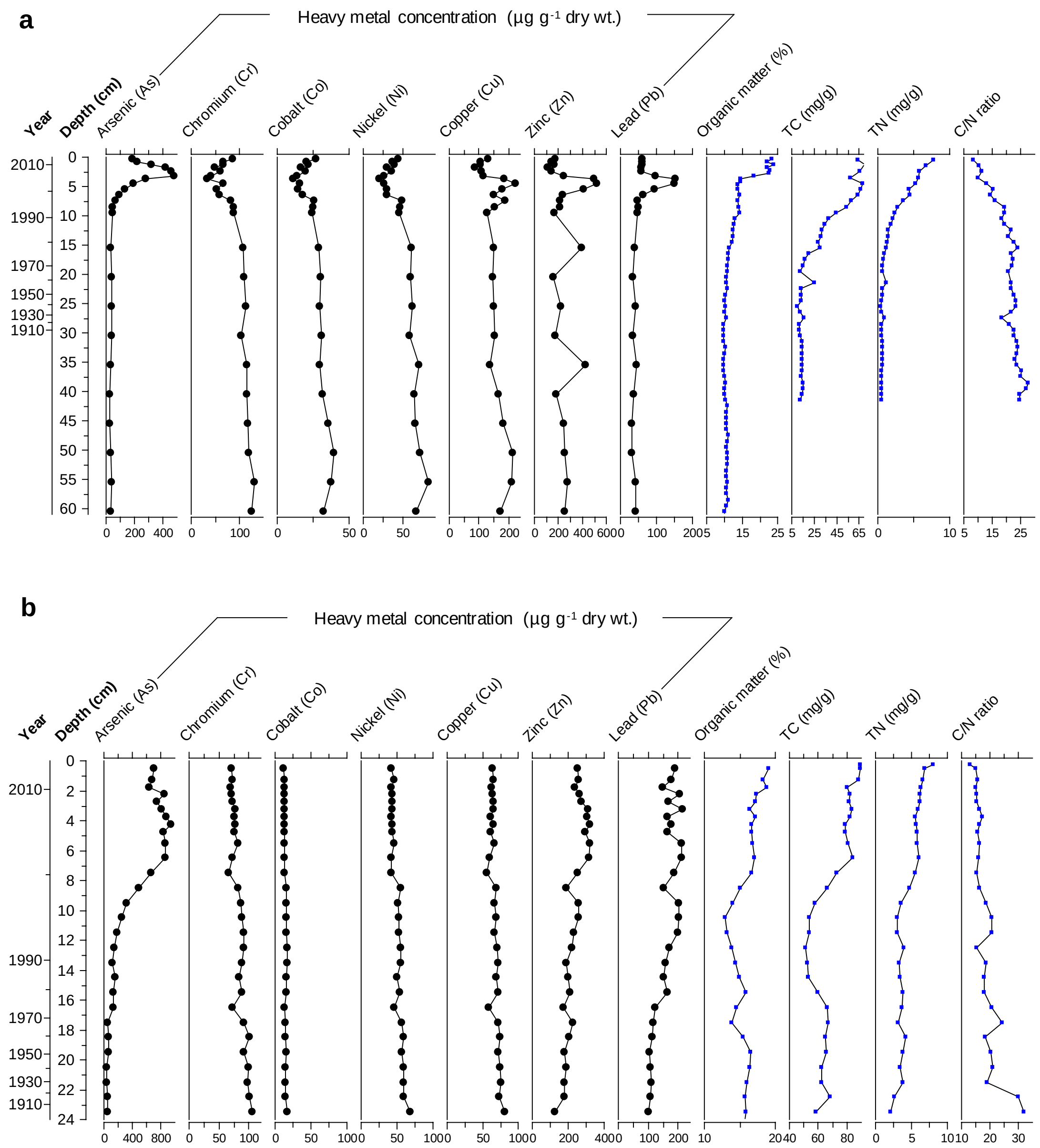

Supplementary Fig. 1. Sedimentary geochemical profiles for Yangzong (**a**) and Datun (**b**) lakes. The changes in seven heavy metal concentrations (arsenic, chromium, cobalt, nickel, copper, zinc, lead), as well as sedimentary organic matter (LOI 550), total carbon (TC), total nitrogen (TN) and atomic C:N ratio, are shown. Note that the scales for heavy metal concentrations are different at each lake. The chronological sequence in calendar years is estimated from the age-depth model constructed for each lake (Supplementary Fig. 4).

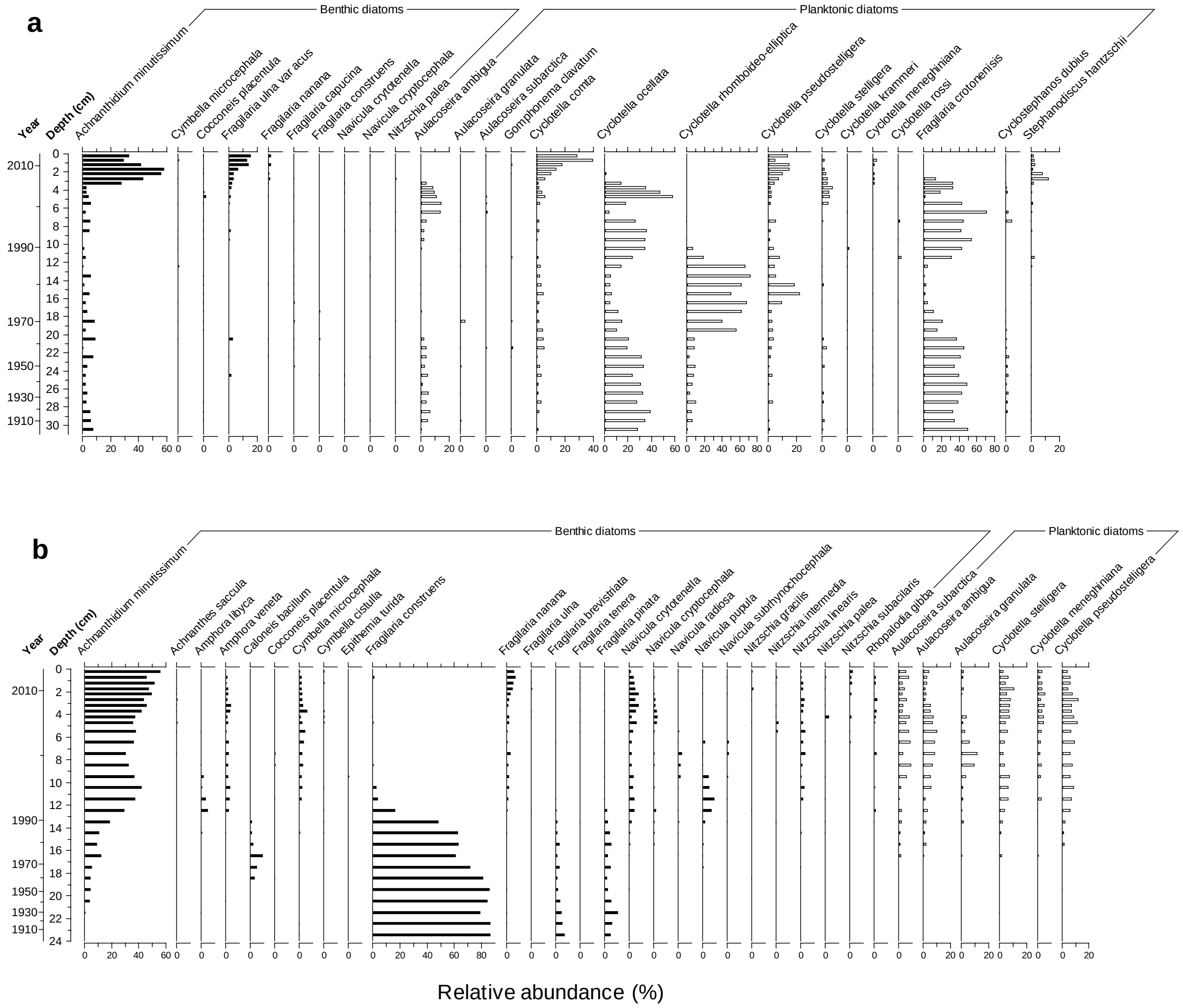

Supplementary Fig. 2. Relative abundance profiles of dominant diatoms (>2%) for Yangzong (**a**) and Datun (**b**) lakes. Diatom habitat category was applied following previous studies [1,2].

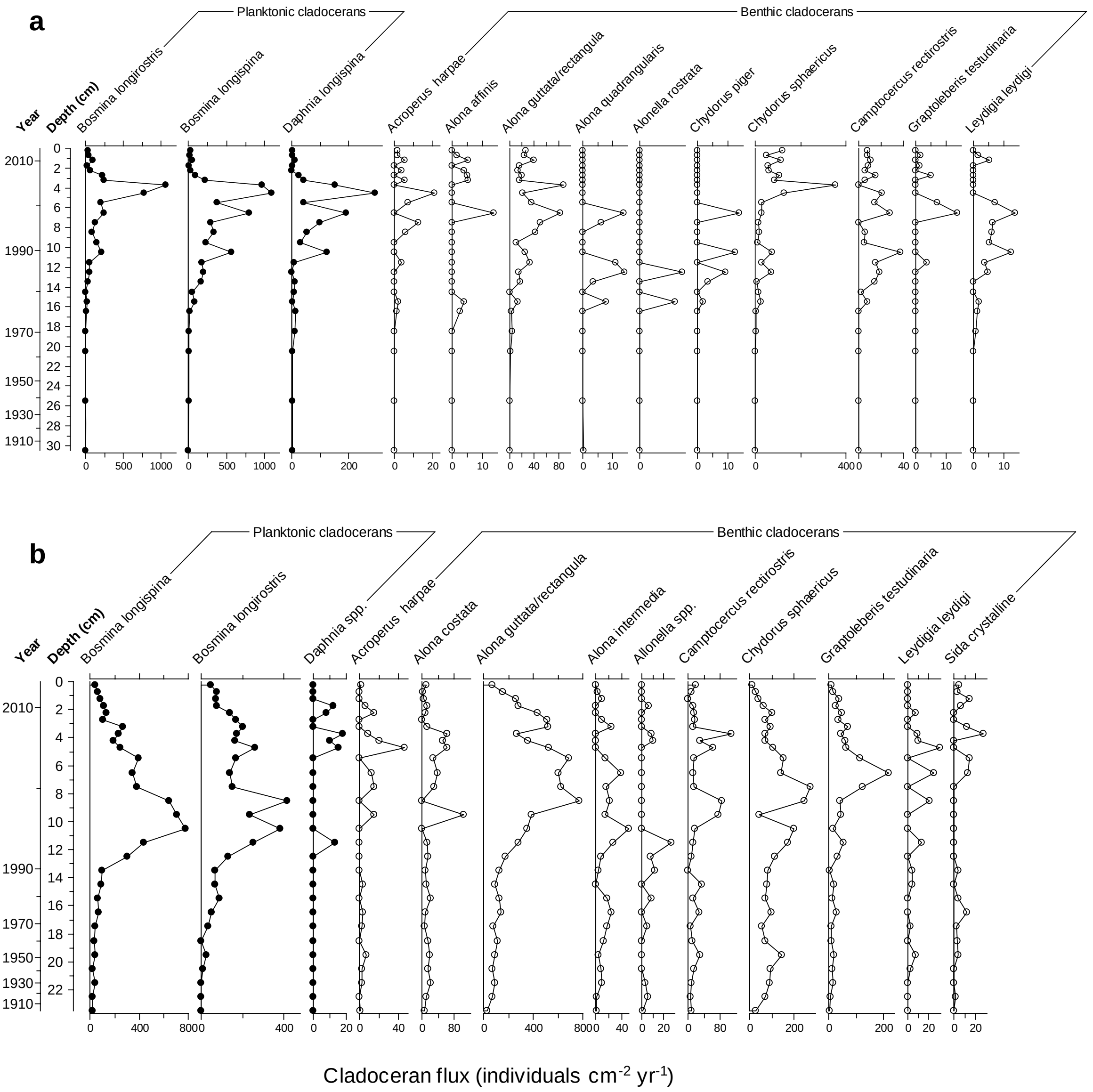

Supplementary Fig. 3. Flux profiles of cladocerans for Yangzong (**a**) and Datun (**b**) lakes.

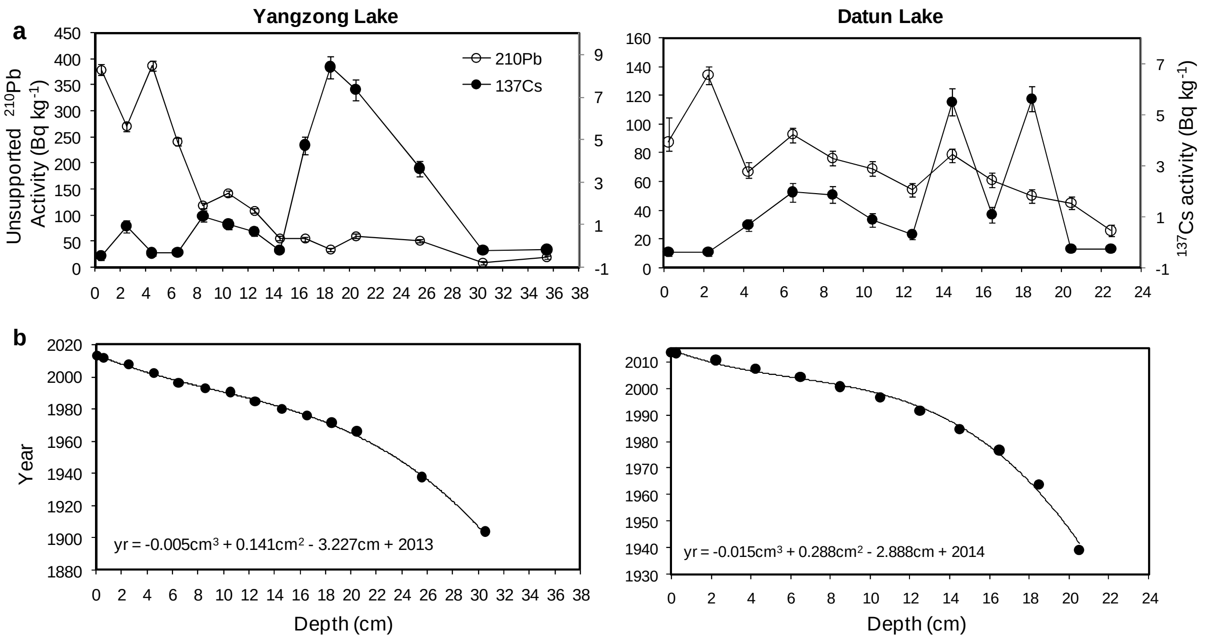

Supplementary Fig. 4. Radioisotopic activities of unsupported ^{210}Pb and ^{137}Cs (±1 SD) for the two lake sediment cores used in this study (**a**). The unsupported ^{210}Pb activities for the sediment intervals for each core were used in developing the constant rate of supply (CRS) models and a third-order polynomial regression with the intercept set as the year of coring was applied to estimate the age (in calendar years) for each sample midpoint (**b**). The mean ^{226}Ra activity (±1 SD) for the sediment cores of Yangzong and Datun was 43.0±7.2 and 44.8±11.8 Bq kg^{-1}, respectively.

Supplementary Table 1. Summary of key limnological features for the two study lakes. Water samples were collected in April (Yangzong Lake, n=8) and July (Datun Lake, n=2) of 2013, respectively, and analyzed by the Water Chemistry Lab of Yunnan Institute of Environmental Sciences, Kunming, China.

	Yangzong	Datun
Latitude (°N)	24°54.42	23°26.77
Longitude (°E)	103°0.41	103°18.71
Altitude (m, a.s.l.)	1,770.8	1,286.0
Catchment area (km^2)	192.0	284.5
Surface area (km^2)	31.7	12.3
Maximum depth (m)	30.0	5.0
Mean depth (m)	19.5	3.7
pH	8.1	9.0
Secchi depth (m)	2.2	0.4
Chlorophyll *a* ($\mu g\ L^{-1}$)	9.3	35.0
Total phosphorus ($\mu g\ L^{-1}$)	38.8	145.0
Total nitrogen ($\mu g\ L^{-1}$)	557.5	1595.0
NO_3^- ($\mu g\ L^{-1}$)	88.0	120.0
NO_2^- ($\mu g\ L^{-1}$)	19.6	4.0
NH_4^+($\mu g\ L^{-1}$)	167.4	225.0
Cl^- ($mg\ L^{-1}$)	10.2	68.5
SO_4^{2-} ($mg\ L^{-1}$)	57.9	158.5
Ca ($mg\ L^{-1}$)	27.2	79.7
Mg ($mg\ L^{-1}$)	29.0	35.2
Na ($mg\ L^{-1}$)	6.0	42.3
K ($mg\ L^{-1}$)	3.9	23.3
Fe ($\mu g\ L^{-1}$)	41.4	24.0
Mn ($\mu g\ L^{-1}$)	4.6	41.5
As ($\mu g\ L^{-1}$)	54.3	213.5
Cr^{6+} ($\mu g\ L^{-1}$)	4.0	4.0
Pb ($\mu g\ L^{-1}$)	9.5	16.5
Cd ($\mu g\ L^{-1}$)	1.1	2.5

Supplementary References

1 Round, F. E., Crawford, R. M. & Mann, D. G. *Diatoms: Biology and Morphology of the Genera*. (Cambridge Univ. Press, 1990).

2 Van Dam, H., Mertens, A. & Sinkeldam, J. A coded checklist and ecological indicator values of freshwater diatoms from The Netherlands. *Aquat. Ecol.* **28**, 117-133 (1994).
